# On-Demand Cueing Sensitive to Step Variability: Understanding Its Impact on Gait of Individuals With Parkinson’s Disease

**DOI:** 10.1109/JTEHM.2025.3563381

**Published:** 2025-04-24

**Authors:** Priya Pallavi, Ankita Raghuvanshi, Suhagiya Dharmik Kumar, Niravkumar Patel, Manasi Kanetkar, Rahul Chhatlani, Manish Rana, Sagar Betai, Roopa Rajan, Uttama Lahiri

**Affiliations:** Department of Electrical EngineeringIndian Institute of Technology Gandhinagar 242275 Gandhinagar 382355 India; Aksharkrupa Hospital Ahmedabad 380004 India; Faculty of Design, Architecture and BuildingUniversity of Technology Sydney 1994 Ultimo NSW 2007 Australia; Design and Innovation CentreIndian Institute of Technology Gandhinagar 242275 Gandhinagar 382355 India; Department of PhysiotherapyMarwadi University Rajkot 360003 India; Sola Hospital Ahmedabad 380060 India; Ansa Clinic Ahmedabad 380054 India; AIIMS New Delhi 110029 India

**Keywords:** On-demand cueing, wearable, freezing of gait, Parkinson’s disease, variability of step time

## Abstract

Parkinson’s disease (PD) is characterized by gait disturbances with freezing of gait (FoG) being one of the most disabling symptoms. The FoG episode is often preceded by an increase in variability in Step Time. As the disease progresses, such gait impairment may become resistant to pharmacotherapy. Use of external cues is an alternative. Existing solutions deliver external cues in a continuous manner that might cause habituation effects, thereby emphasizing the need for on-demand cueing. Manual on-demand cueing upon freezing has been shown to be powerful in bringing an individual out of a freezing state. This can be achieved if one’s proneness to freeze before entering into freezing state can be sensed, and in-turn triggering an external cue on-demand. Motivated by this, we have developed a wearable device ( $\mathrm{SmartWalk}_{\mathrm {VC}}$) that can sense such proneness based on variability in Step Time to offer a visual cue on-demand. We conducted a study involving 20 age-matched healthy individuals and those with PD who walked overground while wearing SmartWalkVC operated in three modes with regard to offering visual cue, namely (a) On-demand cueing, (b) Continuous cueing and (c) No cueing. The results of our study showed that with on-demand cueing, those with PD had minimum variability of Step Time among all the three modes unlike healthy individuals whose gait remained majorly unaffected by different cueing modes. Also, walking speed increased along with a reduction in FoG episodes for those with PD in the on-demand cueing mode compared with the other two modes.Clinical and Translational Impact Statement: Wearable SmartWalkVC quantifies one’s Step Time variability to offer visual cue on-demand, reducing one’s Freezing of Gait that can have clinical significance and be translated to impact one’s social presence.

## I. Introduction

Parkinson’s disease (PD) is a chronic and progressive neurodegenerative disorder affecting approximately 1% of the population over the age of 60 years globally [1]. This is characterized by a range of motor symptoms, including bradykinesia, tremor, etc. with freezing of gait (FoG) being one of the most disabling symptoms [2]. The occurrence of FoG is defined as a sudden, short-duration episode where patients are unable to move their feet forward despite their intention to walk. These episodes can increase with task complexity, such as dual-tasking [3], thereby leading to increased risk of falls [4] that severely compromise the safety and mobility of the affected individuals. Thus, it is important to adopt preventive measures to mitigate the occurrence of such FoG episodes impairing one’s gait.

Pharmacotherapy has been reported as being effective in addressing such impairment of PD patients. However, as the disease progresses, gait impairment may become resistant to medication [5], thereby necessitating alternate solutions [6]. Alternatively, the use of external cueing, such as visual, auditory, and somatosensory (tactile) cues [5] has been reported in literature. Visual cue has emerged as a promising alternative given the ease of perception [7] and has been reported to significantly improve temporal indices of gait [8], such as Step Time which is the time taken between two consecutive steps measured in terms of time elapsed between two consecutive heel-strike events while walking [9]. Conventionally, researchers have explored use of visual cue in terms of using parallel lines marked on the floor [5], or inverted walking stick with protrusion [5], etc. Again, there are researchers who have explored use of technology-assisted visual cueing methods, such as use of walking cane projecting a laser line on the floor [10], Virtual Reality based system displaying a line on the virtual floor [11], shoes projecting a laser line on the floor [12], etc. While both the currently-existing conventional and technology-assisted solutions are powerful in addressing gait impairment of PD patients yet these existing solutions deliver external cue in a continuous manner and are not sensitive to individualized needs. Again, continuous cueing though shown effective in some cases [5], [11], [12] can lead to habituation over prolonged use [13] and can increase one’s cognitive load, thereby emphasizing the need for cue delivery on-demand that is analogous to one receiving assistance only when needed [14]. In fact, literature indicates the use of on-demand cueing offered through manual intervention [14]. However, besides requiring an accompanying individual to trigger the external cue manually that can limit one’s independent navigation, such on-demand cueing is delivered once one has entered the freezing state [14] that disturbs the rhythmicity of gait. Additionally, researchers have used wearable motion sensing technology [15] to deliver cues upon detection of freezing of gait (FoG) episode. However, these systems deliver cues in a reactive manner, i.e., reacting to freezing once it has started, instead of in a predictive manner while taking an action before the freezing starts. This emphasizes the need to get an estimate of one’s proneness to freeze before he / she has entered the freezing state and use a strategy to trigger an external cue on-demand, thereby possibly preserving the rhythmicity of gait or at least minimizing adverse effects on gait rhythmicity. Such an estimation is possible given that the FoG does not occur abruptly [16]. Instead, it is often preceded by a period referred to as the “pre-FoG window” [17] characterized by specific attributes of gait. One of the attributes is an increase in variability in Step Time [9] that has been reported to be an early indicator of forthcoming FoG [18]. Also, such variability in Step Time has been reported to be accompanied by sudden changes in walking speed [19].

Given the importance of delivering an on-demand external cue before freezing happens and the possibility of sensing such a demand based on the variability in one’s Step Time, we wanted to understand the potential of such on-demand cueing vis-à-vis the continuous cueing and absence of cueing (i.e., no cues) on the gait of individuals with PD. For this, we developed a wearable device ( $\mathrm{SmartWalk}_{\mathrm {VC}}$) that can quantify variability in one’s Step Time and use this information to decide on the instant of triggering a visual cue in an individualized manner. We hypothesize that the SmartWalk_VC_ system offering visual cue when needed (On-demand) will contribute to improving the gait of individuals with PD irrespective of the walking task of varying difficulty by at least reducing the number of FoG episodes, reducing the variability in Step Time and improving the walking speed more than when offering visual cue continuously. Thus, our objectives were to (i) design a wearable device ( $\mathrm{SmartWalk}_{\mathrm {VC}}$) that can sense the variability of one’s Step Time during an overground walk and operate in three modes with regard to offering visual cue, namely (a) On-demand cueing, (b) Continuous cueing and (c) No cueing modes, (ii) conduct a study in which one walked overground while wearing SmartWalk_VC_ for comparative investigation of the effect of the three cueing modes on (a) the variability in Step Time of individuals with PD and their healthy counterparts, (b) walking speed and the number of FoG episodes of individuals with PD followed by (iii) taking feedback on the experience of the individuals with PD while walking with the different cueing modes.

This paper is organized as follows: the methodology and setup used for the study are presented in Section II, followed by our observations and findings, which are presented in Section III. Finally, Section IV summarizes the findings, discusses the limitations and the future scope.

## II. Material and Method

### A. System Design

The wearable system ( $\mathrm{SmartWalk}_{\mathrm {VC}}$) comprised of (i) Instrumented Insoles, (ii) Central Module integrated with two peripherals, namely (iii) Visual Cue Unit and (iv) Pathway Segment Detection Unit.

#### 1) Instrumented Insoles

Each of the Instrumented Insoles (placed inside the shoes) had three Force Sensitive Resistors (FSRs). Two FSRs were positioned below the heel (FSR_Heel_ specifically at the lateral and medial locations to take care of any possible inversion / eversion [19] and one FSR was positioned below the toe ( $\mathrm{FSR}_{\mathrm {Toe}}$). The 0 V – 5 V analog signals from the FSRs were acquired by a Central Module along with time stamping.

#### 2) Central Module

The SmartWalk_VC_ had a Microcontroller-based Central Module having a 64 GB SD card (from SanDisk Ultra) as the Data Storage unit. This Module was mounted on a waist belt, similar to that as shown in [20] and integrated with peripheral devices, namely Visual Cue Unit (Section II-A.3) and a Pathway Segment Detection Unit (Section II-A.4). The Central Module of SmartWalk_VC_ housed a Strategy Generator which decided the instant the Visual Cue Unit to be triggered by performing real-time gait analysis.

### B. Real-Time Gait Analysis

**FIGURE 1.**
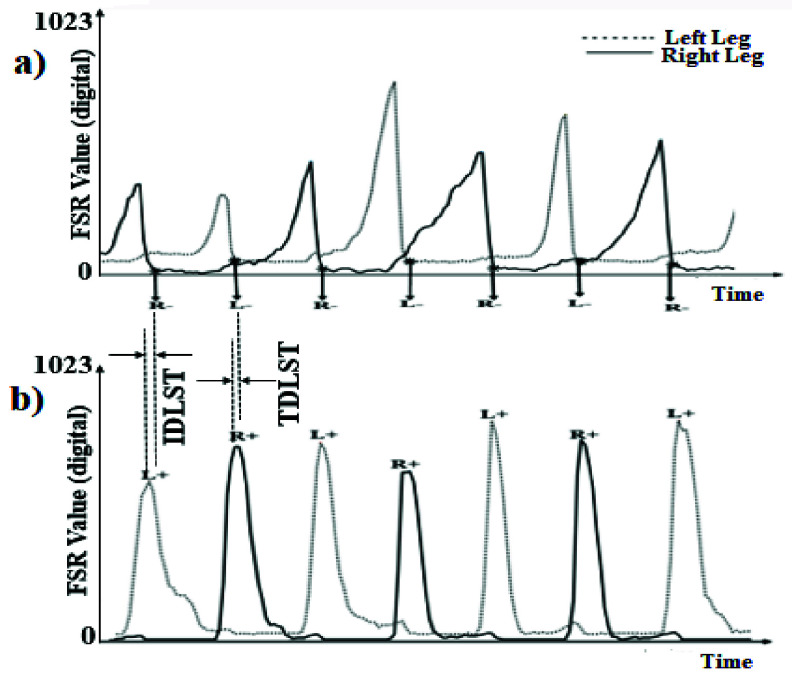
Extraction of Gait Events, namely (a) Toe-off and (b) Heel-strike. Note: ‘IDLST’: initial double limb support time; ‘TDLST’: terminal double limb support time for left leg.

The data acquired from the Instrumented Insoles was used to identify one’s gait events, such as heel-strike and toe-off. Each heel-strike event (labelled as ‘L+’ and ‘R+’ (Fig. 1(b)) for the left and right leg, respectively) and heel-off event was identified from the time stamp corresponding to the valid peak and valley point of the data registered using FSR_Heel_. A peak and valley point was considered as valid if the magnitude of the digital equivalent of the data from FSR_Heel_ (on a scale of 0-1023) was $\ge 50$ units and 0 unit, respectively which were decided based on a pilot study [20] with 10 healthy (Mean (SD)=58(±6.72) years) individuals and 10 individuals with gait disorders (Mean (SD)=62(±4.27) years) walking overground while wearing the Instrumented Shoes. Again, each toe-off event was detected from the time stamp corresponding to the valley point (registered by the FSR_Toe_ in Fig. 1(a)) immediately after the valid peak reflecting the heel-strike event. These events were labelled as ‘L-’ and ‘R-’, representing toe-off events for left and right legs, respectively. The heel-strike and toe-off events were subsequently used to compute gait-related indices, namely Variability in Step Time, and Walking Speed.

#### 1) Computation of Variability in Step Time

Step Time is the duration between consecutive heel-strike events of contralateral legs [9]. The SmartWalk_VC_ used the information on the heel-strike events to compute the Step Time. Subsequently, this information was used to compute variability in Step Time in terms of Coefficient of Variation (%CV) [20] of the Step Time using Eq.(1). We chose this measure since increased variability in Step Time while walking can be one of the indicators of possible freezing [18].

\begin{equation*} \% CV=\frac {Standard~Deviation}{Mean}\ast 100 \tag {1}\end{equation*}

#### 2) Computation of Walking Speed

The SmartWalk_VC_ was used to evaluate the Walking Speed by recording the duration one took to traverse an intermediate 10-meter straight pathway from instants one walked across the pathway delineator placed at 0 m (i.e., START point) and 10 m (i.e., END point) (described below in Section II-A.4).

*2.2 Strategy Generator for Delivery of Visual Cue in an “On-demand cueing” Mode*

**FIGURE 2.**
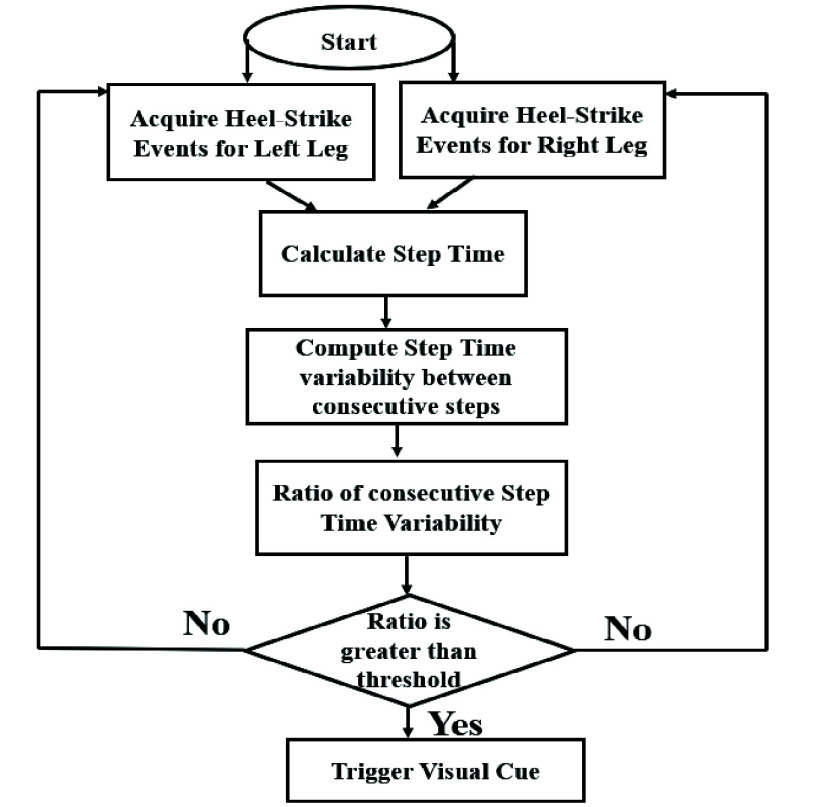
Data flow diagram of strategy generator of SmartWalk_VC_.

The Central Module of the SmartWalk_VC_ was equipped with an ability to decide the instant at which the Visual Cue Unit needed to be triggered. Given that increased variability in Step Time while walking can be one of the indicators of freezing [18], here we chose %CV of Step Time for the Strategy Generator to decide the instant of delivering the visual cue. The Fig. 2 shows the Data Flow Diagram of the Strategy Generator. The Strategy Generator first computed the Step Time from heel-strike events of contralateral legs which in turn was used to compute the variability in Step Time (i.e., %CV of Step Time; Eq. 1). These values were stored in the memory of the microcontroller residing in the Central Module as an array such as, [%CV_n_, %CV_n+1_, %CV_n+2_,...] between Step Time for $\mathrm{step}_{\mathrm {n-1}}$ and step_n_, step_n_ and step_n+1_, and step_n+1_ and step_n+2_, and so on, respectively. Subsequently, the Strategy Generator calculated the ratio of the %CV of Step Time (i.e., $\mathrm{Ratio}_{\mathrm {Step Time variability}}$) which was compared against upper ( $\mathrm{Thresh}_{\mathrm {U}}$) computed from another pilot study [20] and lower ( $\mathrm{Thresh}_{\mathrm {L}}$) thresholds. This pilot study (that was video recorded) aimed to estimate the Thresh_U_ based on a pilot study conducted with 14 individuals with PD (mean (std): 64.7 (8.2) years) who walked overground under single and dual task conditions. Given that a progressive decrease in the strides [4] marked by slowness and variability in gait [3] which might be considered as precursor to a freezing episode [3] in individuals with PD, it is important that such signs of gait variability be identified for necessary preventive measures to be adopted to address issues of forthcoming FoG. The recorded video of the pilot study as annotated by a clinician marking the frames (of video) capturing the variability in one’s gait marked by slowness in gait was used to compute the Thresh_U_ in terms of the $\mathrm{Ratio}_{\mathrm {Step Time variability}}$ which was found to be ~2.5. Additionally, to account for possible hesitation while initiating a walk (often seen in individuals with PD [4]) either at the beginning or anywhere intermediate in a pathway, a range of Thresh_L_, namely 0.5< $\mathrm{Ratio}_{\mathrm {Step Time variability}}< 1$ was chosen as an initial approximation with a hope to help the individual to reinitiate the walk. If such variability in gait was identified, the Strategy Generator of SmartWalk_VC_ triggered visual cue in an “On-demand cueing” mode, i.e., as and when required instead of being delivered in a continuous mode. In contrast, if no such variability in gait was detected, the Strategy Generator continued acquiring information on one’s gait.

#### 3) Visual Cue Unit

**FIGURE 3.**
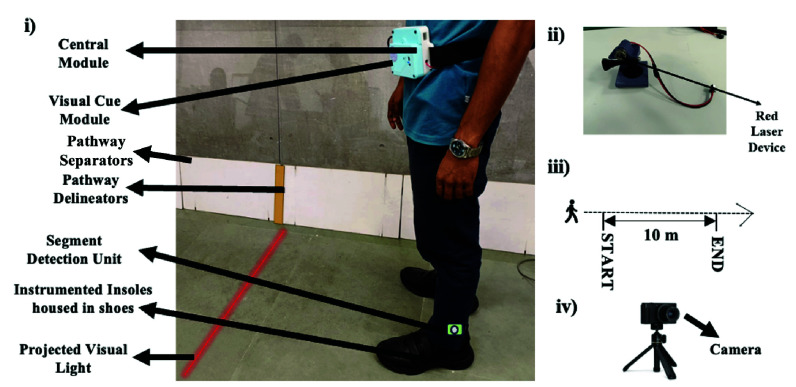
(i) Front view of a person wearing SmartWalk_VC_, (ii) Visual Cue Module of SmartWalk_VC_, (iii) 10m Pathway, (iv) Camera on a tripod.

The Visual Cue Unit of SmartWalk_VC_ (Fig. 3 (i) and (ii)) consisted of a small, lightweight support frame mounted on the waist belt. A laser device (MXD1230 [24]) was attached to adjustable support frame to project a laser line (as a visual cue) of length 1.5m (similar to that as used in another study [21]) on the floor at a distance of *x* m in front of the individual (with *x* being 40% of the height of the individual [22]). The Visual Cue Unit was controlled to operate in the three modes, namely (i) “No cueing” mode ( $\mathrm{Cue}_{\mathrm {None}}$), (ii) “Continuous cueing” mode ( $\mathrm{Cue}_{\mathrm {Cont}}$) and (iii) “On-demand cueing” mode ( $\mathrm{Cue}_{\mathrm {OD}}$). In the Cue_None_ mode, the Visual Cue Unit was kept in the off state with the visual cue being turned off. In contrast, in the Cue_Cont_ and Cue_OD_ modes, the Visual Cue Unit was kept in the on state. However, unlike that in the Cue_Cont_ mode wherein the visual cue was offered continuously irrespective of the decision being taken by the Strategy Generator, in the Cue_OD_ mode, the visual cue was turned on in an “On-demand cueing” mode based on the decision being taken by the Strategy Generator.

#### 4) Pathway Segment Detection Unit

As a part of our present study, we considered a straight pathway with an intermediate stretch of 10m straight segment marked with pathway delineators. For this, we used Ultrasonic Sensor (HCSR04 as in [23]) fitted to the lateral side of one of the Instrumented Shoes. The HCSR04 operates by emitting high-frequency sound waves in a specific direction [24]. When these sound waves encounter a pathway delineator, they are reflected toward the sensor, thereby sending a marker to the Central Module indicating distance of 10m being traversed by the individual.

### C. Experiment and Method

#### 1) Participants

We have designed a study with twenty healthy volunteers (Group_H_; Mean (SD)=64.3 (±9.3) years) and twenty age-matched individuals with PD (Group_PD_; Mean (SD)=65.3 (±9.8) years) (Table 1) recruited from the neighborhood and nearby hospitals, respectively. The inclusion/exclusion criteria for healthy participants were (i) age between 50 and 80 years, (ii) can understand experimenter’s instructions and (iii) have no neurological, musculoskeletal or vestibular impairment. The individuals with PD were checked for their ability to perform 10 m walk-test [25] while walking overground without any external support, e.g., orthosis, canes, and other aids. Additionally, prior to the study, all the participants with PD reported that they experience FoG. The Table 1 shows the clinical measures, such as the Unified Parkinson Disease Rating Scale motor part (UPDRS III) and Hoehn and Yahr (H&Y) score [26] determined in the ‘OFF’ state (for overnight withdrawal of dopaminergic medication for at least 12 hour to ensure occurrence FoG episodes [2]) along with the disease onset duration for the Group_PD_. The study had institute ethical clearance (Approval No.: IEC/UL/2021/024).

**TABLE 1:** Participants’ characteristics.

Healthy Group		Parkinson’s Group				
ID	Age (Yrs.)/ Gender	ID	Age (Yrs.)/ Gender	UPDRS III	H&Y Score	DOD (years)
H1	75/M	P1	76/M	15	2	2
H2	74/M	P2	75/M	16	2	2
H3	52/F	P3	50/F	21	2	4.5
H4	68/F	P4	68/F	15	3	2
H5	52/F	P5	53/F	17	2	2
H6	69/M	P6	70/M	20	3	2
H7	70/M	P7	72/M	15	3	3
H8	50/M	P8	50/M	23	2	7
H9	52/M	P9	52/M	15	2	1
H10	55/M	P10	55/M	40	3	10
H11	58/M	P11	60/M	15	2	9
H12	70/M	P12	72/M	28	3	4
H13	60/F	P13	62/F	18	2	8
H14	75/M	P14	74/M	40	3	7
H15	75/M	P15	76/M	50	3	13
H16	70/M	P16	71/M	26	2	7
H17	75/M	P17	78/M	44	3	2
H18	69/M	P18	72/M	20	3	2
H19	52/F	P19	54/F	28	2	3
H20	65/F	P20	67/F	14	2	4
Avg. (SD)	64.3 (9.3)	Avg. (SD)	65.3 (9.8)	23.9 (11.0)	2.45 (0.5)	4.75 (3.4)

Note: ID: Participants’ ID, Yrs: Years, UPDRS III: Unified Parkinson’s Diseases Rating Scale motor part, H&Y: Hoehn and Yahr, DOD: Disease Onset Duration as per medical record.

#### 2) Experimental Setup

The experimental setup (Figs. 3 (i) and (ii)) included (i) SmartWalk_VC_ comprising of Instrumented Insoles housed in a pair of shoes and a Central Module along with peripherals, namely (ii) Visual Cue Unit mounted on the waist belt and a Pathway Segment Detection Unit mounted on the lateral side of one of the shoes, (iii) a straight pathway with an intermediate stretch of 10m straight segment marked with pathway delineators (Fig. 3 (iii)) and (iv) a camera held on a tripod.

#### 3) Procedure

Here, we conducted a study that needed a commitment of ~40 minutes from each participant. When the participant entered the study room, the experimenter showed the experimental setup and told the participant that the task needed him / her to walk overground on a straight pathway while the device ( $\mathrm{SmartWalk}_{\mathrm {VC}}$) would be operated in three modes, such as three visual cueing modes Cue_None_, Cue_Cont_ and Cue_OD_ and describing each mode. Also, the participants were told that they were expected to walk under Single Task ( $\mathrm{Task}_{\mathrm {S}}$) and Dual Task ( $\mathrm{Task}_{\mathrm {D}}$) conditions, similar to that in literature [27]. In the Task_S_ condition, one was expected to walk at a self-selected (comfortable) speed without speaking. Again, in the Task_D_ condition, one was expected to walk on the pathway at a self-selected (comfortable) speed while holding a tray in his/her hand. Also, the experimenter informed the participant that he/she was free to discontinue from the study at any time in case of inconvenience. When the participant showed willingness to participate, the accompanying clinician (in the team) collected the demographic details of the participants and obtained the clinical measures, such as UPDRS-III and H&Y scores. This was followed by signing of the consent form. Once the participant expressed that he / she was ready to start the walk, the experimenter helped the participant to wear the SmartWalk_VC_. Further, the participant was told to stand upright before commencing the walk. This was followed by the participant walking on a straight pathway having an intermediate stretch of 10 m labeled using pathway delineators and Segment Detection Unit (Fig. 3)) under any of the two task conditions (Task_S_ and $\mathrm{Task}_{\mathrm {D}}$), with SmartWalk_VC_ being operated in any one of the three cueing modes (Cue_None_, Cue_Cont,_ and $\mathrm{Cue}_{\mathrm {OD}}$). Though each participant walked on a longer stretch of pathway that was more than 10 m, yet we recorded the data for the intermediate 10 m segment of the straight pathway. The order of presentation of the task condition and cueing mode was randomized across the participants in order to eliminate any ordering effect [27]. A camera was used to video-record the walk of each participant. The video was used for subsequent analysis. Specifically, this was used by a clinician to label segments of the video either as ‘segment demonstrating FoG’ (i.e., occurrence of FoG episodes) or ‘segment demonstrating no FoG’ (i.e., absence of FoG episodes). Furthermore, the experimenter collected feedback from each participant belonging to the Group_PD_ at the end of each walking trial. In this, each participant was asked “How was your experience while walking when you did not see any line on the floor in front of you?” ( $\mathrm{Q}_{\mathrm {Cue\_None}}$), “How was your experience while walking when you could see a line on the floor in front of you before every step you took?” ( $\mathrm{Q}_{\mathrm {Cue\_Cont}}$) and “How was your experience while walking when you could intermittently see a line on the floor in front of you?” ( $\mathrm{Q}_{\mathrm {Cue\_OD}}$). The responses were reported using the Likert Scale ranging from 1 (Very Bad) to 5 (Very Good).

#### 4) Computation of Correlation Coefficient

To understand the clinical relevance of the effect of On-demand cueing on the freezing of gait episodes vis-à-vis that of continuous cueing and no cueing modes, we carried out Pearson’s Correlation test [28] using (2) between the number of freezing episodes as labeled by the clinician with duration of disease onset and UPDRS-III scores of individuals with PD.

\begin{align*} & Correlation ~Coefficient \\ & = \frac {\sum \nolimits _{i=1}^{N} {(x_{i}-\overline x )(y_{i}-\overline y )}}{\sqrt {\sum \nolimits _{i=1}^{N} {(x_{i}-\overline x )}^{2} \sqrt {\sum \nolimits _{i=1}^{N} {(y_{i}-\overline y )}^{2}}}} \tag {2}\end{align*}

Here, x_i_ represents the number of freezing episodes, y_i_ is the duration of disease onset and UPDRS-III scores of individuals with PD, ‘i’ is the participant ID, $\bar {x}$, $\bar {y}$ are the sample means for x_i_ and y_i_ respectively, and ‘N’ is the total number of individuals with PD.

#### 5) Statistical Analysis

Here, we used IBM SPSS Statistics 20 [29]software for statistical analysis. First, the ANOVA test [29] was done on the gait parameters measured by using the SmartWalk_VC_ system and then the residuals were tested for normality using the Shapiro-Wilk [29]. Since the data was not normally distributed, we used non-parametric tests. To understand the effect of the three cueing modes on the gait performance of participants belonging to the two groups, i.e. Group_H_ and Group_PD_, we did an independent group sample analysis using the Mann-Whitney test [29]. Furthermore, to investigate the within-group effects of cueing modes on the gait parameters, we did a dependent sample analysis using the Wilcoxon-signed-rank test [29].

## III. Results and Discussion

Here we present our results based on the data captured by our system when the Healthy group ( $\mathrm{Group}_{\mathrm {H}}$) and the group with Parkinson’s Disease ( $\mathrm{Group}_{\mathrm {PD}}$) walked overground on a straight pathway under Single ( $\mathrm{Task}_{\mathrm {S}}$) and Dual ( $\mathrm{Task}_{\mathrm {D}}$) Task conditions while wearing SmartWalk_VC_ programmed to operate in three different cueing modes e.g., (i) “No cueing” ( $\mathrm{Cue}_{\mathrm {None}}$), (ii) “Continuous cueing” ( $\mathrm{Cue}_{\mathrm {Cont}}$) and (iii) “On-demand cueing” that was sensitive to Step Time Variability ( $\mathrm{Cue}_{\mathrm {OD}}$).

### A. Comparative Investigation of the Effect of on-Demand Cueing, No Cueing and Continuous Cueing of $\text{SmartWalk}_{VC}$ on Step Time Variability of Two Participant Groups for Overground Walk Under Single and Dual Task Conditions

We wanted to understand whether the various cueing modes (Cue_OD_, Cue_None_ and $\mathrm{Cue}_{\mathrm {Cont}}$) can (i) have differentiated implications on one’s Step Time variability and (ii) contribute to the improvement in gait in terms of reducing the Step Time variability.

#### 1) Comparative Analysis for Healthy Group

**FIGURE 4.**
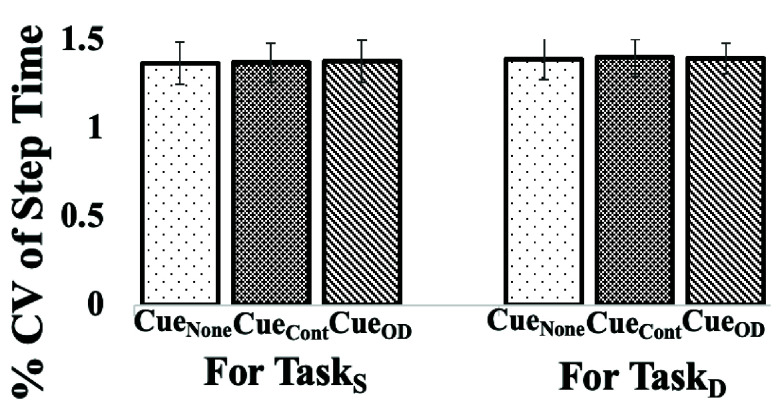
Comparative Group Analysis of % CV of Step Time for (a) Single ( $\mathrm{Task}_{\mathrm {S}}$) and (b) Dual ( $\mathrm{Task}_{\mathrm {D}}$) Task Conditions for Healthy Elderly Group. Note: Cue_None_: “No cueing” mode, Cue_Cont_: “Continuous cueing” mode and Cue_OD_: “On-demand cueing” mode sensitive to Step Time Variability.

Fig. 4 shows the Step Time variability for the Healthy Group ( $\mathrm{Group}_{\mathrm {H}}$) while walking on the pathway for both task conditions (Task_S_ and $\mathrm{Task}_{\mathrm {D}}$) under different cueing modes (Cue_OD_, Cue_None_ and $\mathrm{Cue}_{\mathrm {Cont}}$) of SmartWalk_VC_. It can be seen that Group_H_ demonstrated nearly similar %CV of Step Time irrespective of the cueing modes. Specifically, the change in the %CV of Step time across cueing modes was minimal ( $\Delta=0.33$%, 0.83% and 0.50% between Cue_None_ & Cue_Cont_, Cue_None_ & Cue_OD_ and Cue_Cont_ &Cue_OD_, respectively under Task_S_; and $\Delta=0.40$%, 0.20% and 0.20% between Cue_None_ & Cue_Cont_, Cue_None_ & Cue_OD_ and Cue_Cont_ &Cue_OD_, respectively under $\mathrm{Task}_{\mathrm {D}}$). Given that variations in Step Time are related to one’s walking speed [30], we analyzed their walking speed which was found to remain nearly the same across the cueing modes and the task conditions (For details, please see Section III-B below). Such an observation may reflect the rhythmicity in the healthy gait [30] that remains significantly unaffected by the cueing modes and irrespective of the task conditions.

#### 2) Comparative Analysis for Group with Parkinson’s Disease

**FIGURE 5.**
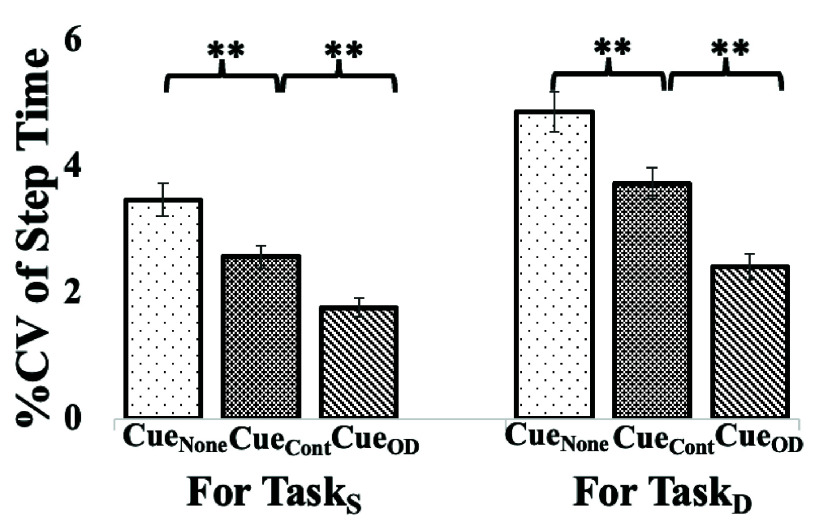
Comparative group analysis of average % CV of Step Time under(a) Single (Task_S_) and (b) Dual (Task_D_) Task Conditions for Group withParkinson’s Disease. Note: Cue_None_: “No cueing” mode, Cue_Cont_: “Continuous cueing” mode andCue_OD_: “On-demand cueing” mode sensitive to Step Time Variability,**: pvalue<0.001.

It can be seen from Fig. 5 that for the group with Parkinson’s Disease ( $\mathrm{Group}_{\mathrm {PD}}$), the %CV of Step Time changed significantly across the cueing modes, unlike the Healthy Group (Fig. 4). Irrespective of the Single and Dual Task conditions, we see a reducing trend in the %CV of Step Time from Cue_None_ to Cue_Cont_ to Cue_OD_ with the %CV of Step Time being minimum for Cue_OD_ among all the cueing modes.

Specifically, the change in %CV of Step Time between Cue_None_ and Cue_Cont_ modes was 26.04% and 23.37%, under Task_S_ and Task_D_, respectively. Again, the change in %CV of Step Time between Cue_None_ and Cue_OD_ modes was 49.25% and 50.57% under Task_S_ and Task_D_, respectively. Finally, the change in %CV of Step Time between Cue_Cont_ and Cue_OD_ modes was 31.38% and 35.49% under Task_S_ and Task_D_, respectively. All these changes were statistically significant (p-value<0.001). Again, unlike the healthy group, for Group_PD_, we could see significant variation in the walking speed across the cueing modes and the task conditions (For details, please see Section III-B below).

The minimum Step Time variability under Cue_OD_ mode for Group_PD_ might suggest the importance of delivering cues as and when needed instead of offering continuously which might be associated with additional cognitive load in attending to the cue continuously along with the intrusiveness and habituation effect reducing the gait efficiency [13].

### B. Understanding the Effect of on-Demand Cueing in Comparison with No Cueing and Continuous Cueing Modes on Walking Speed of Two Participant Groups for Overground Walk Under Single and Dual Task Conditions

**FIGURE 6.**
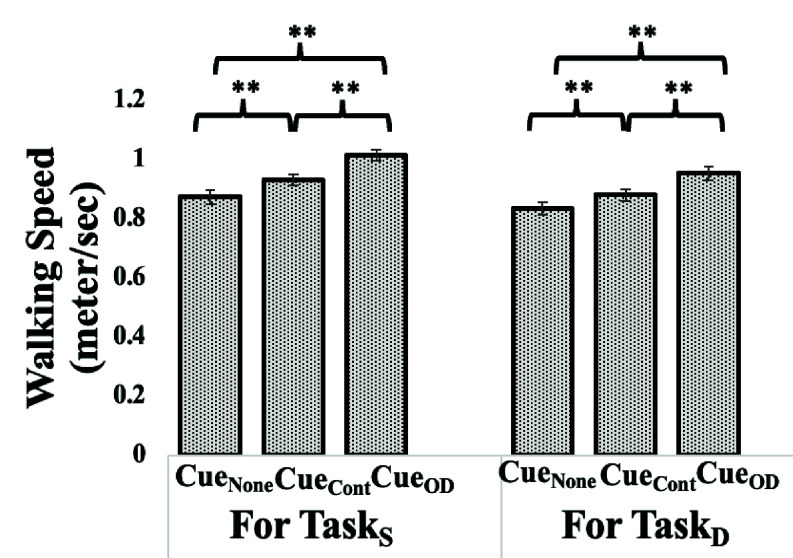
Comparative group analysis of average walking speed under (a)Single (Task_S_) and (b) Dual (Task_D_) Task Conditions for Group withParkinson’s Disease. Note: Cue_None_: “No cueing” mode, Cue_Cont_: “Continuous cueing” mode andCue_OD_: “On-demand sensitive to Step Time Variability cueing” mode;**:p-value<0.001.

Having seen that the “On-demand cueing” ( $\mathrm{Cue}_{\mathrm {OD}}$) triggered by the SmartWalk_VC_ was powerful in contributing to statistical reduction in the %CV of Step Time compared with Cue_Cont_, particularly for the Group_PD_ and the fact that Step Time is related to one’s walking speed along with slow walking speed being one of the hallmarks of gait of individuals with PD [19], we wanted to investigate the implication of Cue_OD_ vis-à-vis that for Cue_None_ and Cue_Cont_ on the Walking Speed of Group_PD_ under single task and dual task conditions. From Fig. 6, we can see that irrespective of the task conditions, there was an increasing trend in the Walking Speed of Group_PD_ from Cue_None_ to Cue_Cont_ to Cue_OD_, with the group average Walking Speed being maximum for the Cue_OD_ mode among all the cueing modes. Also, the increase in Walking Speed from Cue_None_ to Cue_Cont_ modes ( $\Delta=6.66$%, 5.34% for Task_S_ and Task_D_, respectively), Cue_None_ to Cue_OD_ modes ( $\Delta=16.22$%, 14.15% for Task_S_ and Task_D_, respectively) and Cue_Cont_ to Cue_OD_ modes ( $\Delta=8.96$%, 8.37% for Task_S_ and Task_D_, respectively) were statistically significant (p-value<0.001). In contrast, we found no significant change in walking speed of the Group_H_ for Task_D_ compared to Task_S_. Their average walking speed in Task_S_ was 1.08 m/s, 1.06 m/s and 1.07 m/s for Cue_None_, Cue_Cont_, and Cue_OD_ modes, respectively. Again, their average walking speed in Task_D_ was 1.03 m/s, 0.99 m/s and 1.05 m/s for Cue_None_, Cue_Cont_, and Cue_OD_ modes, respectively. The observed average gait speeds (0.99–1.08 m/s) were slightly lower than expected for healthy older adults [31], potentially due to our participant composition (35% of participants belonging to Group_H_ were under 60 years of age and 60% of the Group_H_ were above 60 years of age; Table 1). Again, such low average gait speeds might be attributed to the task environment wherein the participants were under observation by the experimenter causing the older adults to demonstrate slower walking that is in line with literature [32].

In short, for the Group_PD_, our findings suggest that the “On-demand cueing” emerged as the most powerful among all the cueing modes in both the task conditions in terms of enabling this group to walk overground at higher Walking Speed than even the Cue_Cont_ mode, possibly inferring improved gait of this group in the Cue_OD_ mode, similar to that seen from the reduction in the %CV of Step Time (Section III-A.2).

### C. Understanding the Effect of on-Demand Cueing in Comparison with No Cueing and Continuous Cueing Modes on Freezing of Gait of Individuals with Parkinson’s Disease

Given that the main aim was to ensure uninterrupted gait of individuals with PD, we wanted to understand whether the “On-demand cueing” could contribute to any reduction in freezing of gait of Group_PD_ in terms of (i) the number of freezing episodes and (ii) duration of freezing episodes if any when compared with that in presence of Cue_Cont_and Cue_None_.

**FIGURE 7.**
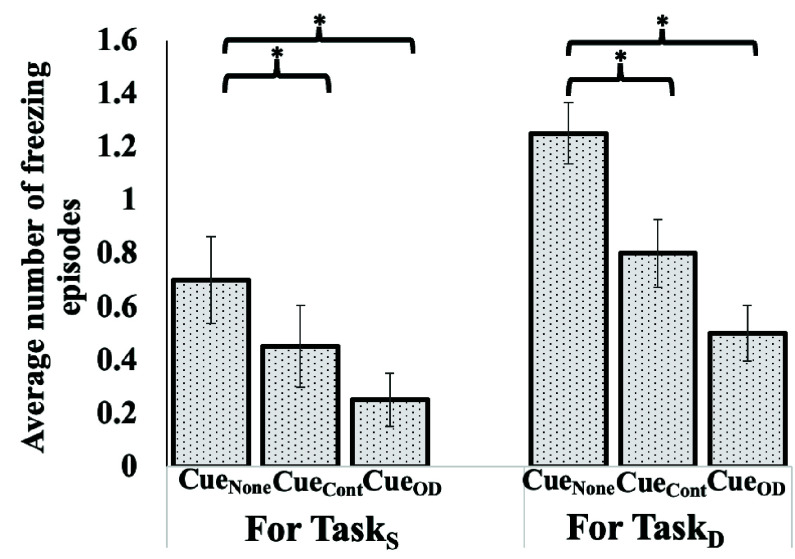
Comparative group analysis of average number of freezing of gaitepisodes under (a) Single (Task_S_) and (b) Dual (Task_D_) Task Conditions forGroup with Parkinson’s Disease. Note: Cue_None_: “No cueing” mode, Cue_Cont_: “Continuous cueing” mode andCue_OD_: “On-demand sensitive to Step Time Variability cueing” mode, *:p-value<0.05.

With regard to the number of freezing episodes, it can be seen from Fig. 7 that among the three cueing modes, the number of FoG episodes was the minimum for the Cue_OD_ mode irrespective of the task conditions, though not statistically significant when compared to that with Cue_Cont_. In fact, in the on-demand cueing mode, the SmartWear_VC_ offered visual cues for 36 (accounting for $9.94\pm 4.56$% of the total walking duration) and 45 (accounting for $12.54\pm 6.29$% of the total walking duration) times in total for all the participants combined, respectively for Task_S_ and Task_D_.

Again, with regard to the duration of the freezing episodes as evident from the video segments labeled by the clinician (Section II-B), we saw that on an average, the duration an individual froze as a part of the walking duration was the least for Cue_OD_ mode among all the three modes irrespective of the task condition. Specifically, for Task_S_ condition, on an average, the participants froze for $11.16\pm 3.88$%, $10.19\pm 3.82$%, and $5.04\pm 2.20$% of the total walking duration for Cue_None_, Cue_Cont_, and Cue_OD_ modes, respectively. Also, for Task_D_condition, the participants froze for $16.51\pm 4.35$%, $14.84\pm 4.03$%, and $9.11\pm 2.89$%of the total walking duration for Cue_None_, Cue_Cont_, and Cue_OD_ modes, respectively.

Though our findings on the percentage time frozen of our participants with PD are found to be in line with the findings reported in other studies [33], [34], [35] where individuals with PD were in the OFF state, yet the relatively higher values of the time frozen by the participants with PD in our study might be attributed to both the OFF state (with respect to medication) of our participants, and the task environment particularly with regard to the pathway used in our study. Specifically, the straight pathway used in our study was an overground stretch that was demarcated as a pathway using pathway separators to simulate a footpath walking zone of around 1.8 meters in width [36] having intermediate pathway delineators (Fig. 3). Such a pathway might have been viewed by our participants with PD as a restricted passageway contributing to higher percentage time frozen in line with findings from literature [37].

### D. Understanding the Clinical Significance of the Effect of on-Demand Cueing on Freezing of Gait Episodes

To understand the clinical relevance of the effect of On-demand cueing on the freezing of gait episodes vis-à-vis that of continuous cueing and no cueing modes, we computed the correlation (Section II-B.4) of the number of freezing episodes with duration of disease onset and UPDRS-III scores (Table 1) across different cueing conditions and task type.

In the Cue_OD_ mode, the number of freezing episodes correlated weakly with the disease onset duration (with r =0.30 and 0.39, for Task_S_ and Task_D_ respectively; [28]) and moderately with the UPDRS-III (with r =0.58 and 0.65, for Task_S_ and Task_D_ respectively; [28]). In contrast, in the Cue_None_ and Cue_Cont_ modes, the number of freezing episodes correlated strongly with both the disease onset duration (with r =0.70 and 0.71, respectively [27]) and UPDRS-III scores (with r =0.76 and 0.85, respectively [27]) in Task_S_. Similarly, the number of freezing episodes correlated strongly with the disease onset duration in the Cue_None_ mode (with r =0.70; [27]) and moderate-to-strongly in the Cue_Cont_ mode (with r =0.63; [27]) and strongly with UPDRS-III in the Cue_None_ mode (with r =0.81) and Cue_Cont_ mode (with r =0.82; [27]) in Task_D_.

Given the small sample size, though we do not intend to generalize our findings, yet an overall reduction in the association of the number of freezing episodes with disease onset duration and UPDRS-III scores under the Cue_OD_ mode vis-à-vis that under Cue_Cont_ and Cue_None_ modes reflects the potent of the Cue_OD_ mode to help this target group manage freezing episodes that can possibly have clinical relevance.

### E. Patients’ Feedback: Investigating the Preference of the Cueing Modes for Individuals with Parkinson’s Disease

We wanted to understand the participants’ experience while walking under the three modes of cueing delivered by our SmartWalk_VC_ system. To achieve this, we gathered post-study feedback in response to $\mathrm{Q}_{\mathrm {Cue\_None}}$, $\mathrm{Q}_{\mathrm {Cue\_Cont}}$ and $\mathrm{Q}_{\mathrm {Cue\_OD}}$ (Section II-B.3) from each participant belonging to Group_PD_. Feedback was collected after exposing the participant to each of the cueing modes, i.e., Cue_OD_, Cue_None_ and Cue_Cont_.

**FIGURE 8.**
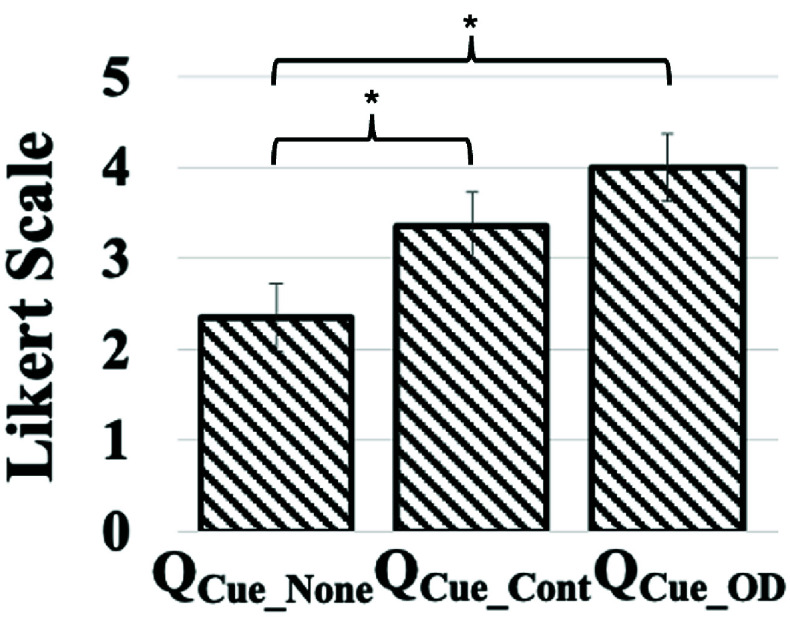
User Feedback on different cueing modes provided usingSmartWalk_VC_. Note: Score ranging from 1 (Very Bad) to 5 (Very Good); QCue_None_: “Howwas your experience while walking when you did not see any line on thefloor in front of you?”; QCue_Cont_: “How was your experience while walkingwhen you could see a line on the floor in front of you before every step youtook?”; QCue_OD_: “How was your experience while walking when you couldintermittently see a line on the floor in front of you?”; p-value<0.05.

The results (Fig. 8) show that the participants’ experience while walking under the Cue_None_ mode received the lowest average rating. In contrast, the participants’ experience improved on receiving cues while walking than that in which no cue was delivered. Specifically, the ratings for $\mathrm{Q}_{\mathrm {Cue\_None}}$ were significantly less than each for $\mathrm{Q}_{\mathrm {Cue\_Cont}}$ (with p<0.05) and $\mathrm{Q}_{\mathrm {Cue\_OD}}$ (with p<0.05). Though no significant differences were found between $\mathrm{Q}_{\mathrm {Cue\_Cont}}$ and $\mathrm{Q}_{\mathrm {Cue\_OD}}$, participants rated Cue_OD_ the highest highlighting a preference for on-demand cueing. When asked the reason behind their preference for getting a cue while walking, all the participants said that taking a step ahead was easier for them in the presence of a line projected on the floor in the front. Among the Cue_Cont_ and Cue_OD_ modes, the participants’ experience was the best for the Cue_OD_ mode. In fact, when asked the reason behind their improved experience while walking with Cue_OD_ mode than the Cue_Cont_mode, most of them said that they felt that whenever they were slowing down and were thinking of getting external assistance, the system was able to understand their problem and they could see a line in front of them which helped them to take a step forward.

## IV. Conclusion

In our present work, we have developed SmartWalk_VC_ as a wearable device which can be programmed to operate in three different modes namely (i) “No cueing” mode ( $\mathrm{Cue}_{\mathrm {None}}$), (ii) “Continuous cueing” mode ( $\mathrm{Cue}_{\mathrm {Cont}}$) and (iii) “On-demand cueing” mode ( $\mathrm{Cue}_{\mathrm {OD}}$) with regard to offering visual cue. The Cue_OD_ was sensitive to variability in Step Time, which was sensed by our wearable device. Our aim was to investigate the effect of “On-demand cueing” on the gait of individuals with PD who walked overground on a straight pathway under varying task conditions and understand its significance while comparing the effect with that of the other two modes. Results of our study showed that with the on-demand cueing, those with PD had minimum variability of Step Time among all the three modes unlike healthy individuals whose gait remained majorly unaffected by different cueing modes. Also, walking speed increased along with reduction in FoG episodes for those with PD in the on-demand cueing mode compared with the other two cueing modes. This suggests that the Cue_OD_ mode might hold promise in terms of facilitating gait of individuals with PD as this mode of cueing effectively reduced FoG episodes, enhancing gait continuity and stability marked by lower variability in the steps being taken, indicating a more consistent walking pattern along with increased gait speed.

Though our results are promising, our study had certain limitations. One of the limitations was limited exposure and limited sample size, with single exposure to each of the three cueing modes being offered to each participant. In future, we plan to extend this study in which participants will be offered multiple exposures to each of the “Continuous cueing” and “On-demand cueing” modes wherein we expect that the difference in the number of FoG episodes between the two modes will be pronounced given the element of effect of habituation being felt on repeated exposure, particularly to the “Continuous cueing”. Although our present study was conducted outside lab-based/clinic setting, further investigation in real-world settings involving patients with more advanced stages of disease is required for broader applicability. Again, although in our present study, we had access to the UPDRS-III and H&Y scores of our participants with PD, we did not have access to other clinical measures, such as Levodopa Equivalent Daily Dose (LEDD) [38] and New Freezing of Gait Questionnaire (N-FOGQ) [39] which we plan to acquire in future studies. Again, given that the focus of our present study was on understanding the potential of “On-demand cueing” mode on the gait of individuals with PD suffering from FoG vis-à-vis other cueing modes, we used only one type of cue, i.e., visual cue. This opens up avenue to see the effect of other types of cues, such as auditory and tactile cues offered in the “On-demand cueing” mode on the gait of individuals with PD that we plan to do in the future.

Notwithstanding the limitations, the findings of our study lay a stepping-stone to help design future studies aimed to answer deeper questions on the role of cueing mode and its effect on gait of individuals with PD. Also, our SmartWalk_VC_ offers a wearable solution to quantify gait and address issues related to FoG that has clinical relevance. The measured gait-related indices can offer complementary information that can be useful for taking clinical decisions with an overall aim to improve gait of individuals with PD along with improved navigation ability in social settings.
